# The influence of alcohol consumption on Self-Rated Health and Mood during the COVID-19 pandemic in Spain

**DOI:** 10.3389/fpubh.2023.1257459

**Published:** 2023-10-10

**Authors:** Raquel Sánchez-Recio, Juan Antonio Parrilla-Huertas, Ángela Asensio-Martinez, Sergio Valdivieso-Pardos, María Zúñiga-Antón, Marta Cerdán-Bernad

**Affiliations:** ^1^Research Group on Health Services in Aragon (GRISSA), Department of Preventive Medicine and Public Health, University of Zaragoza, Zaragoza, Spain; ^2^Institute for Health Research in Aragon (IIS Aragón), Zaragoza, Spain; ^3^Department of Geography and Spatial Planning, University of Zaragoza, Zaragoza, Spain; ^4^Aragonese Primary Care Research Group (GAIAP), Department of Psychology and Sociology, University of Zaragoza, Zaragoza, Spain; ^5^Territory, Society and Geographic Visualization, Zaragoza City Council, Zaragoza, Spain; ^6^Spatial Planning Study Group, Department of Geography and Spatial Planning, University of Zaragoza, Zaragoza, Spain; ^7^General Secretariat of Penitentiary Institutions, Zuera, Spain

**Keywords:** alcohol-consumption, pandemic, Self-Rated Health, Mood, moderation

## Abstract

**Introduction:**

There is evidence of a significant upturn of certain unhealthy lifestyle choices such as Alcohol Consumption during the COVID-19 pandemic.

**Objective:**

To analyze whether Alcohol Consumption has increased since the onset of pandemic and whether it affects the relationship between Mood and Self-Rated Health among adult Spanish population.

**Methodology:**

Study of two cross-sectional cohorts (1—initial period of confinement COVID-19 pandemic, 2—between fifth and sixth waves of pandemic) to examine Alcohol Consumption in the relationship between Mood and Self-Rated Health using a moderation analysis with PROCESS macro for SPSS.

**Results:**

5,949 people (62.1% women) participated in the study. Alcohol Consumption showed a significant increase, with men consuming more Alcohol than women in both periods (58.6% vs. 44.7% and 72.1% vs. 56.7%, respectively, *p* < 0.001). The moderation analysis revealed that sex and Alcohol Consumption conditioned the relationship between Mood and Self-Rated Health in the first survey, with a greater effect on women, who stated that not consuming Alcohol had a positive effect on the relationship between Mood and Self-Rated Health (B: −0.530; *p* < 0.001).

**Discussion:**

Currently it is about of implementing strategies to manage the pandemic—some of them aimed at promoting healthy living and stress management as assets that favor healthy lifestyles with fewer risk factors. New studies are needed to address the social thresholds of alcohol consumption, considering different perspectives for understanding variations in the intrapersonal and social perception of drunkenness, as this has been shown to be inconsistent across cultures and time periods.

## 1. Introduction

In 2020, the rapid spread of the COVID-19 pandemic forced all countries to take measures to contain the advance of the disease and prevent the oversaturation of their healthcare systems. One of the most drastic measures was confining the population in their homes, which created an exceptional and unfamiliar situation (1). Anxiety and concern rose due to the lack of information about the possible consequences, the potential loss of employment, the economic difficulties, the increased vulnerability and inequalities, misinformation and the fear of the unknown (1–4).

During the first period of strict confinement, as a coping strategy to deal with social isolation, many people engaged in activities at home such as exercise, cooking, watching TV or using the internet (5–7). However, people also developed unhealthy behaviors, such as overuse of alcohol and anxiolytics, online gaming and greater consumption of snacks or unhealthy meals (8–10), to try to reduce the stress they were experiencing (11, 12).

Several studies have reported an increase in alcohol sales in supermarkets during the period of strict confinement (13, 14). In Spain, a study by the Ministry of Agriculture, Fisheries and Food revealed that, during strict confinement, beer consumption rose by 86.5%, wine by 73.4%, and spirits by 93.4% (15). Several authors, such as Mota et al. and Testino, have opened a debate on whether the psychological consequences of confinement were a risk factor for alcohol consumption and on the relationship of this consumption with the decrease in people’s immunity and the increase in health complications during the COVID-19 pandemic (16, 17).

Alcohol and tobacco are related to one another, as they are usually consumed together (13). During the pandemic, there was a surge in tobacco use and in relapses among former smokers (18–20). This fact, together with alcohol consumption at home, has been associated with higher risk of addiction for the other members of the family, especially children (21–23).

Stress and other disorders, especially mental disorders (with a prevalence of 33% in adults) also increased during the pandemic. In Spain, the survey on mental illness published in 2021 showed that 22% of the people surveyed during COVID-19 reported more depressive disorders than before the outbreak. In addition, in 2021 the Ministry of Health revealed that the consumption of antidepressants in Spain amounted to 4.05 pills per 1,000 inhabitants. Recent research in Spain by Del González-López et al. (24) on the use of psychiatric medication during the COVID-19 pandemic has showed that the increase in the use of this type of medication was especially significant among women, adults and people living in rural areas.

At the same time, mood and anxiety disorders have been strongly linked to poor Self-Rated Health and the mediating role of community belonging and food insecurity between mood and/or anxiety disorders and Self-Rated Health (25–27).

Considering these circumstances and owing to the distinctive characteristics of the pandemic within the Autonomous Community of Aragon, specifically in the city of Zaragoza, it is our belief that modifications in the health-related behaviors of the populace may become evident. Consequently, such changes could subsequently contribute to specific alterations in the way individuals perceive their well-being and emotional condition.

The aims of the present study are, firstly, to describe the consumption patterns of Alcohol, tobacco and anxiolytics and the use of information and communication technologies (ICTs)—such as TV and online gaming—at two points in time during the COVID-19 pandemic (I: confinement and II: 20 months later) in the population of Zaragoza over 16 years and, secondly, to study the influence of Alcohol Consumption on the relationship between Mood and Self-Rated health in the same population at the two aforementioned points in time.

## 2. Materials and methods

A cross-sectional study of the population of Zaragoza aged over 16 years old in two periods (cohorts) during the COVID-19 pandemic. The first period (first cohort) was during the first pandemic wave, in strict confinement (in Spain the period of strict confinement was from 14 March to 21 June 2020). The second period (second cohort), 20 months after the first survey, was from 15 November to 15 December 2021. Zaragoza is a city in southern Europe with 717,259 inhabitants (22).

The anonymized data were obtained from the first online COVID-19 survey,” Living conditions, needs and expectations during the COVID-19 confinement, “and second online COVID-19 survey II” Living conditions, needs and expectations after 20 months of the COVID-19 pandemic,” conducted by the Zaragoza City Council [ENS (28)]. The Zaragoza city council was responsible for obtaining informed consent. The first survey showed the concerns and desires of people living in Zaragoza during home confinement in the harsh, critical months of March to May 2020. The objective of the second survey was to discover the concerns and desires of these citizens after 20 months of pandemic. Both surveys include 52 questions grouped into 5 thematic blocks: (1) Personal situation: (household and family economy); (2) State of mind and health; (3) Activities and attitudes during the pandemic; (4) Post-pandemic horizon; and (5) Assessment of the services of the City Council and other public administrations and services. The samples for both surveys were selected using the snowball method supported by digital marketing techniques. The entire population of Zaragoza over 18 years of age was invited to participate in the two surveys. In order to achieve greater participation, the surveys were distributed through the different information channels of the City Council of Zaragoza, with the participation of the different municipal associations and the University of Zaragoza. Only those surveys that were fully completed were recorded.

### 2.1. Variables

The behaviors that the population developed during the two moments of the COVID-19 pandemic analyzed were described using the following variables of interest (Table 1): Consumption of Alcohol, tobacco, anxiolytics, and use of ICTs (online shopping, watching pay and free TV).

**Table 1:** Description of the variables of the study and the survey questions.

		Question used in the first survey	The question used in the second survey^*^
Sex		What is your sex? Male, Female, I prefer not to answer.	What is your sex? Male, Female, I prefer not to answer.
Outcome variable	Self-rated health	How would you rate your current health [from very good (1) to very bad] (5?)?	How would you rate your current health is [from very good (1) to very bad] (5?)?
Criterion variable	Mood	“How do you feel about yourself before and during the COVID-19 confinement? There are 5 response options: 1 (very bad), 2(bad), 3 (average), 4 (good) and 5 (very good)	“How do you feel about yourself before and during the COVID-19 confinement? There are 5 response options: 1 (very bad), 2(bad), 3 (average), 4 (good) and 5 (very good)
Behavior variables	Alcohol	Do you report having consumed Alcohol during the COVID-19 confinement?	Do you report having consumed Alcohol during the COVID-19 pandemic?
	Tobacco	Do you report having consumed tobacco during the COVID-19 confinement?	Do you report having consumed tobacco during the COVID-19 pandemic?
	Anti-anxiety medication	Do you report having consumed anxiolytics during the COVID-19 confinement?	Do you report having consumed anxiolytics during the COVID-19 pandemic?
	Use of ITCs	Do you report online gaming, online shopping, watching pay and free TV during the COVID-19 confinement?	Do you report online gaming, online shopping, watching pay and free TV during the COVID-19 confinement?
Covariables	Age	What age range are you in? ([16–17 years]; [18–49 years]; [50–64 years]; [65–84 years]; and [85 and over])	What age range are you in? ([16–17 years]; [18–49 years]; [50–64 years]; [65–84 years]; and [85 and over])
	Education level	What is your education level? The response options are as follows: no studies, primary education, compulsory secondary education, upper secondary education or higher-level vocational training and university studies.	What is your education level? The response options are as follows: no studies, primary education, compulsory secondary education, upper secondary education or higher-level vocational training and university studies.
	Income	What is your household monthly income (in Euros)? The response options are as follows: > 1,000 Euros, between 1,000 and 2,000 Euros, between 2,001 and 3,000 Euros, between 3,001 and 4,000 Euros, and > 4,000 Euros.	What is your household monthly income (in Euros)? The response options are as follows: > 1,000 Euros, between 1,000 and 2,000 Euros, between 2,001 and 3,000 Euros, between 3,001 and 4,000 Euros, and > 4,000 Euros.
	Chronic disease	Have you been diagnosed with a chronic disease? Yes, no, no answer.	Have you been diagnosed with a chronic disease? Yes, no, no answer.

^*^In the initial explanation provided prior to administering this online survey, the respondents were informed at all times what specific moment of the pandemic was being referred to. While completing the survey, the respondents were constantly reminded of the point in time to which the questions referred.

Regarding people’s appraisal of their health, the importance of the contextual framework of the literature is well-known (29). The review of the scientific literature shows that Mood modulates people’s responses to how they rate their health (30, 31). Therefore, based on the literature, the following variables of interest were selected for this study: Self-Rated Health as a result variable, Mood as a criterion variable and Alcohol as a moderating variable (Table 1).

The outcome variable (Self-Rated Health) was obtained with the question” How is your health “with 5 response options from 1 (very bad) to 5 (very good). This way of collecting self-reported health is commonly used in numerous health surveys both in Spain (28, 32, 33) and in Europe (34).

The criterion variable (Mood) was obtained with the question “How do you feel about yourself before and during confinement due to COVID-19?” With 5 possible answers from 1 (very bad) to 5 (very good).

Finally, the following sociodemographic variables were considered as covariables:

- Age was recorded in three groups: (i) ([16–49 years]; (ii) [50–65 years]; (iii) [> 65 years]).
- Education level was defined in three groups (35): (i) low (primary and compulsory secondary education); (ii) medium (upper secondary education and intermediate-level vocational training) and (iii) high (high-level vocational training and university studies).
- Income (net monthly income in Euros per household) was classified into: ([<1,000], [1,000–2,000], [2,000–3,000], [3,000–4,000], and [>4,000]).
- Chronic disease: yes or no.

### 2.2. Analysis

**Figure 1:**
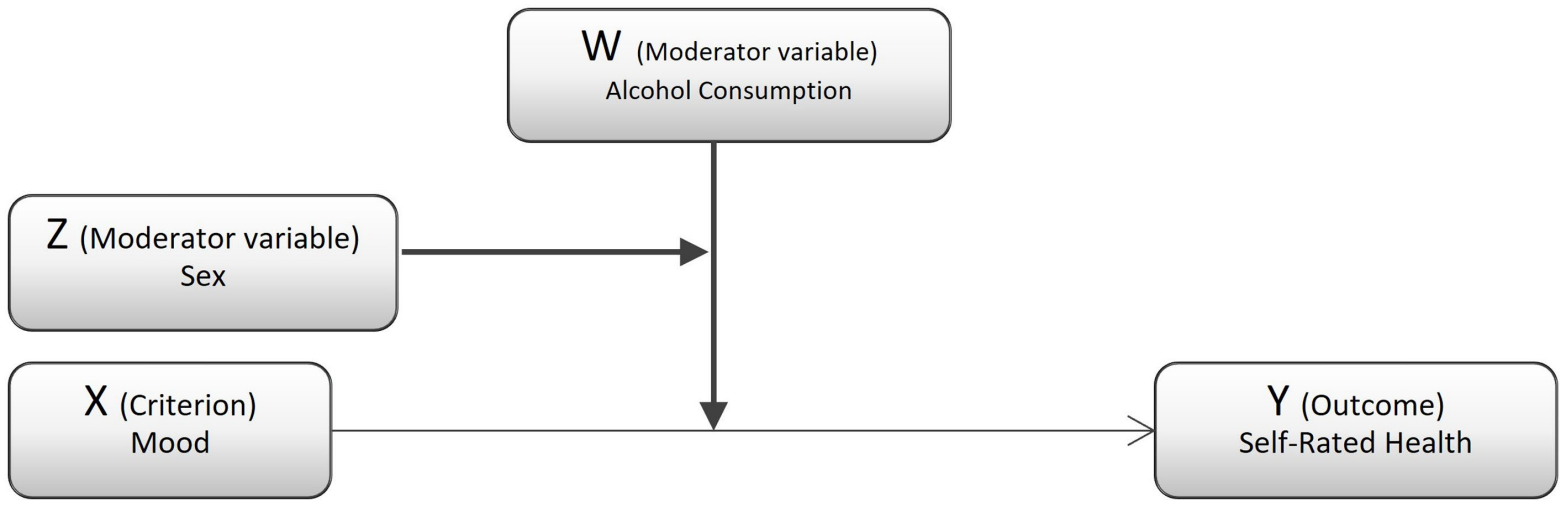
Conceptual diagram of the moderation process.

Initially, withing descriptive statistical analyses, a frequency analysis was used to describe sociodemographic data and general information about the survey participants; their differences were assessed using the Chi-square test. The association between Mood and Self-Rated Health in the study population during two periods in the COVID-19 pandemic analyzed and whether this association was influenced by third variables were examined using ordinary least-squares linear regression analysis. This model serves to statistically evaluate how a criterion variable or causal antecedent exerts an effect on a dependent or consequential variable X − Y. This model can be complemented by third variables that explain the objective more precisely (36, 37) (Figure 1).

The model proposed above was tested using the PROCESS macro with IBM SPSS-24®, model 3, using 10,000 bootstrapped samples. Weighting factors were applied in all the analyses, calculated to avoid errors associated with design or non-response. Statistical significance was set at *p* < 0.05. Once the microdata had been evaluated and filtered by the corresponding municipal body, the City Council of Zaragoza provided them to the Chair of Territorial Planning to be used anonymously.

## 3. Results

### 3.1. Descriptive statistics

The first and second surveys yielded 4,186 and 1,763 valid responses, respectively. The analysis of the sociodemographic characteristics (sex and age) of both samples using the census data available revealed that they contained characteristics like those of the Aragonese society as a whole at the time of the study.

**Figure 2:**
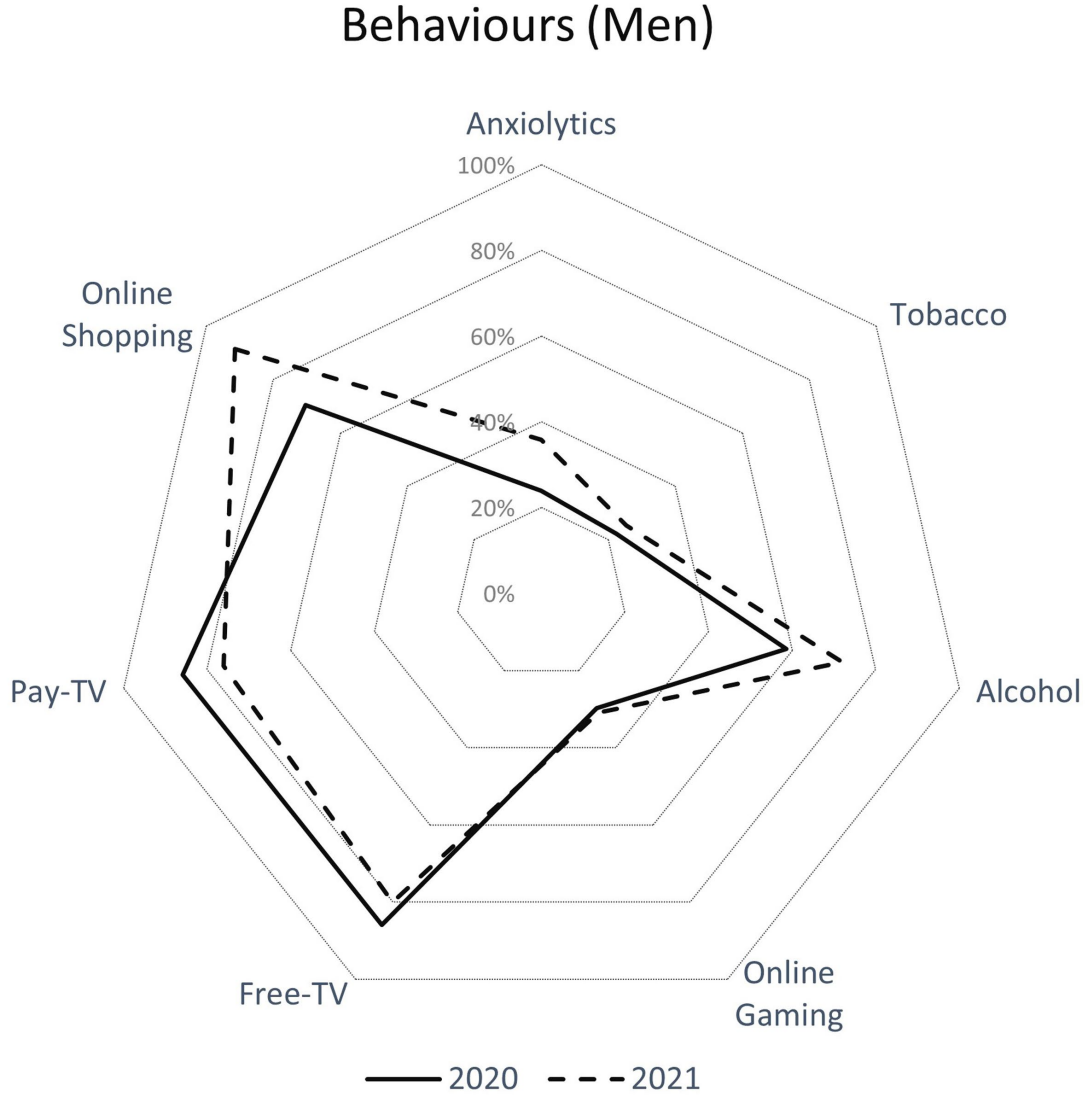
Health behaviors (consumption of anxiolytics, tobacco, alcohol, online gaming, pay and free TV and online shopping) in men during the first and second online COVID-19 surveys in Aragon (Spain).

**Figure 3:**
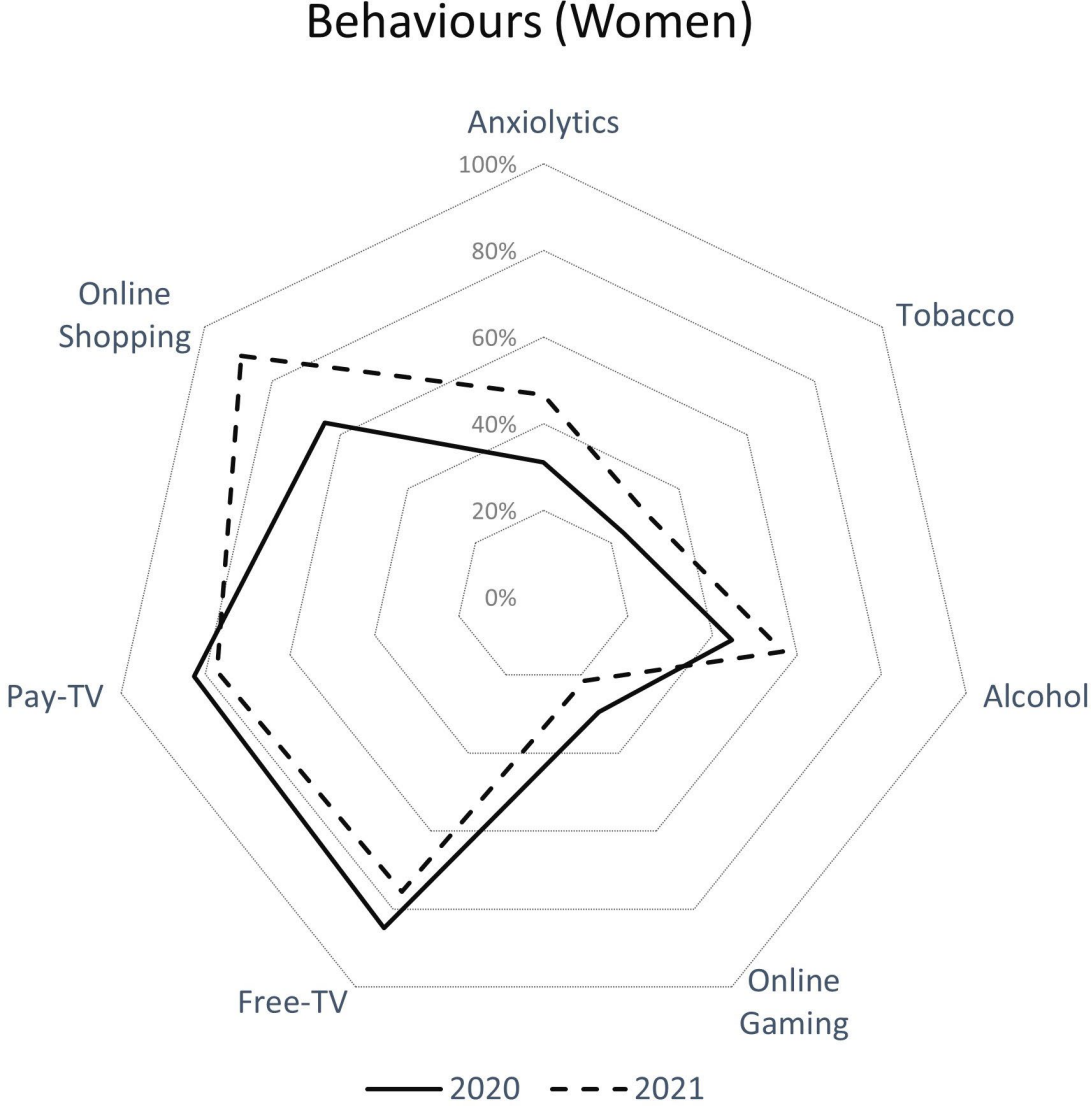
Health behaviors (consumption of anxiolytics, tobacco, alcohol, online gaming, pay- and free-TV and online shopping) in women during the first and second online COVID-19 surveys in Aragon (Spain).

Table 2 and Figures 2, 3 present the sample description of the variables in this study. The most represented population group in both surveys was between 16 and 49 years old in both sexes. Self-Rated Health was worse during the first survey for both men and women than in the second and men reported better Self-Rated Health than women in both surveys. More than 50% of men and women reported having a high education level. Concerning income, in the first survey people mainly reported having a monthly income of 1,000–2,000 Euros (31% men and 35% women) and 2,001–3,000 Euros (34% men and 31% women). In the second survey, people reported the same income per month than in the first survey.

**Table 2:** Sample description of variables.

		2020			2021			
		Men (1,585)	Women (2,601)	p (χ2)	Men (663)	Women (1,100)	p (χ2)	p (χ2) (year of survey)
Variables		*n* (%)	*n* (%)		*n* (%)	*n* (%)		
Age	16 to 49 years	931 (58.73%)	1,815 (69.78%)	<0.001	342 (51.58%)	688 (62.54%)	<0.001	<0.001
	50 to 65 years	540 (34.07%)	691 (26.56%)		246 (37.10%)	364 (33.09%)		
	+65 years	114 (7.19%)	95 (3.65%)		75 (11.31%)	48 (4.36%)		
Education	Low	231 (14.57%)	350 (13.45%)	0.028	76 (11.46%)	153 (13.91%)	0.172	0.025
	Medium	479 (30.22%)	706 (27.14%)		214 (32.27%)	318 (28.91%)		
	High	875 (55.20%)	1,545 (59.40)		373 (56.26%)	629 (57.18%)		
Income	<1,000 Euros/month	92 (5.80%)	271 (10.41%)	<0.001	30 (4.52%)	73 (6.63%)	<0.001	<0.001
	Into 1,000–2,000 Euros/month	492 (31.04%)	911 (35.02%)		141 (21.26%)	385 (35%)		
	2,001–3,000 Euros/month	534 (33.69%)	823 (31.64%)		277 (34.23%)	343 (31.18%)		
	3,001–4,000 Euros/month	301 (18.99%)	399 (15.34%)		175 (26.39%)	194 (17.64%)		
	4,001 Euros/month	166 (10.47%)	197 (7.57%)		90 (13.57%)	105 (9.54%)		
Self-rated health	Very good	319 (20.12%)	477 (18.39%)	0.002	198 (29.86%)	250 (22.73%)	0.029	<0.001
	Good	511 (32.23%)	866 (78.72%)		124 (18.70%)	221 (20.09%)		
	Average	483 (30.47%)	708 (26.01%)		183 (27.60%)	304 (27.64%)		
	Bad	185 (11.67%)	328 (12.61%)		99 (14.93%)	203 (18.45%)		
	Very bad	87 (5.48%)	222 (8.53%)		59 (8.89%)	122 (1.09%)		
Mood	Very good	74 (4.66%)	83 (3.19%)	0.052	57 (8.59%)	59 (5.36%)	0.001	0.004
	Good	335 (21.13%)	525 (20.18%)		155 (23.37%)	189 (17.18%)		
	Average	638 (40.25%)	1,024 (39.36%)		295 (44.49%)	544 (49.45%)		
	Bad	420 (26.49%)	753 (28.9%)		97 (14.63%)	201 (18.27%)		
	Very bad	118 (7.44%)	216 (8.30%)		59 (8.89%)	107 (9.72%)		
Chronic disease	Yes	529 (33.37%)	673 (25.87%)	<0.001	194 (29.26%)	378 (34.36%)	0.015	0.001
	No	1,056 (66.62%)	1,928 (74.12%)		469 (70.73%)	722 (65.63%)		

Mood presented the same pattern than Self-Rated Health, but the latter was worse in the first survey than in the second in both men and women. This perception improved in a statistically significant way in the second survey, but women reported a considerably worse Mood than men in both surveys. In the first survey, 33.37% of men and 25.87% of women reported having a chronic disease; the percentages in the second survey were 29.26% for men and 34.36% for women.

Regarding the health-related behaviors that the population developed (consumption of anxiolytics, tobacco, Alcohol, online gaming, watching pay and free TV) during the two periods analyzed, both sexes (Figure 2, men; Figure 3, women) experienced a rise in the consumption of anxiolytics (men 23.9% vs. 35.7%, women 31.1% vs. 46.7%; *p* < 0.001), tobacco (men 22.3% vs. 25.3%, women 23.6% vs. 30.5%; *p* < 0.001), Alcohol (men 58.6% vs. 72.1%, women 44.7% vs. 56.7%; *p* < 0.001) and online gaming (men 70.4% vs. 91.4%, women 64.5% vs. 89.2%; *p* < 0.001). This increase in health behaviors was greater in women than in men (*p* < 0.001). Finally, both sexes experienced a decrease in pay- and free-TV viewing in the second survey with respect to the first one.

### 3.2. Multiple regression analysis, interpretation of results, and moderation analysis

In this study, Alcohol and sex were used as moderator variables. A moderator variable, commonly denoted as W, is a third variable that affects the strength of the relationship between a predicted variable and predictor variable in a correlation. Multiple regression techniques were used to analyze the direct relationship between Mood and Self-Rated Health. Hayes’ moderation analysis was run to check the moderation effect of Alcohol and sex on the predictor and predicted variables. This process is considered an effective and more powerful processor than the alternatives. This study used 10,000 bootstrap resamples. Bootstrapping does not involve any assumption of normal distribution.

Table 3A shows the results of the multiple regression analysis for the first online COVID-19 survey in Aragon (first pandemic wave) in the period of strict confinement. The results reveal that Mood (B: 0.456, *p* < 0.001) has a positive and affirmative effect on Self-Rated Health (predicted variable). Sex (B: −0.530, *p*:0.001) and age (B: −0.144, *p* < 0.001) have a negative effect on Self-Rated Health. Finally, chronic disease (B: 0.481, *p* < 0.001) and income (B: 0.073, *p* < 0.001) have a positive and significant relationship with Self-Rated Health. The outcomes showed that the regression model is significant. Furthermore, using a Bonferroni correction, Self-Rated Health is associated with Mood (F: 96.0473, *p* < 0.001).

**Table 3:** Moderation analysis: Outcome variable Self-Rated Health, moderator variables sex and Alcohol Consumption (**A**, Survey I, 2020; **B**, second online COVID-19 survey, 2021).

						CI 95%	
		B	se	t	*p*	LLCI	ULCI
**A. Survey I, 2020**							
	Constant	2.756	0.160	17.255	0.000	2.443	3.069
	Mood	0.456	0.046	9.980	0.000	0.367	0.546
Moderation 1	Alcohol consumption	0.145	0.180	0.803	0.422	−0.209	0.498
Moderation 2	Sex	−0.530	0.166	−3.197	0.001	−0.855	−0.205
Covariables	Chronic disease	0.481	0.040	11.903	0.000	0.402	0.560
	Age	−0.141	0.032	−4.464	0.000	−0.203	−0.079
	Education	0.013	0.026	0.514	0.607	−0.038	0.065
	Income	0.073	0.017	4.203	0.000	0.039	0.107
	Int_1 (Mood × Alcohol)	−0.032	0.059	−0.537	0.591	−0.148	0.084
	Int_2 (Mood × Sex)	0.143	0.055	2.596	0.010	0.035	0.251
	Int_3 (Alcohol × Sex)	0.448	0.228	1.966	0.049	0.001	0.895
	Int_4 (Mood × Alcohol × Sex)	−0.148	0.076	−1.952	0.051	−0.296	0.001
	**R**	**R-sq**	**MSE**	**F**	**df1**	**df2**	***p***
	0.4528	0.2050	1.2634	96.0473	11	4.097	0.0000
**B. Second online COVID-19 survey, 2021**							
	Constant	2.202	0.360	6.125	0.000	1.497	2.907
	Mood	0.327	0.066	4.956	0.000	0.197	0.456
Moderation 1	Alcohol consumption	−0.572	0.389	−1.470	0.142	−1.334	0.191
Moderation 2	Sex	−0.411	0.377	−1.089	0.276	−1.150	0.329
Covariables	Chronic disease	0.529	0.073	7.229	0.000	0.385	0.672
	Age	−0.077	0.056	−1.377	0.169	−0.186	0.033
	Education	0.068	0.052	1.306	0.192	−0.034	0.169
	Income	0.162	0.034	4.766	0.000	0.095	0.229
	Int_1 (Mood × Alcohol)	0.090	0.080	1.135	0.257	−0.066	0.246
	Int_2 (Mood × Sex)	0.059	0.079	0.741	0.459	−0.096	0.213
	Int_3 (Alcohol × Sex)	0.413	0.475	0.869	0.385	−0.519	1.344
	Int_4 (Mood × Alcohol × Sex)	−0.069	0.100	−0.690	0.490	−0.264	0.127
	**R**	**R-sq**	**MSE**	**F**	**df1**	**df2**	***p***
	0,4,538	0,2,059	1,9,811	41,2,823	11	1,751	0,0000

Dependent variable, Self-Rated Health; LLCI, lower limit confidence interval; ULCI, Upper limit confidence interval; CI95%, Confidence interval 95%; Int., Interaction.

Table 3B presents the results of the multiple regression analysis for the second online COVID-19 survey in Aragon (sixth wave in Spain). The results reveal that Mood (B: 0.327, *p* < 0.001) has a positive effect on Self-Rated Health (predicted variable). Chronic disease (B: 0.529, *p* < 0.001) and income (B: 0.161, *p* < 0.001) have a positive and significant relationship to Self-Rated Health. The outcomes showed that the regression model is significant. Furthermore, using a Bonferroni correction, Self-Rated Health is associated with Mood (F: 41.2823, *p* < 0.001).

In Table 3A, Interaction 2 (Mood × Sex) has a significant effect on Self-Rated Health with *p*:0.010. Interaction 3 (*p*:0.049) and Interaction 4 (0.051) have a significant effect on Self-Rated Health. Finally, Table 3B shows that no interaction has a significant effect on Self-Rated Health.

### 3.3. Interpretation of plotting

**Figure 4:**
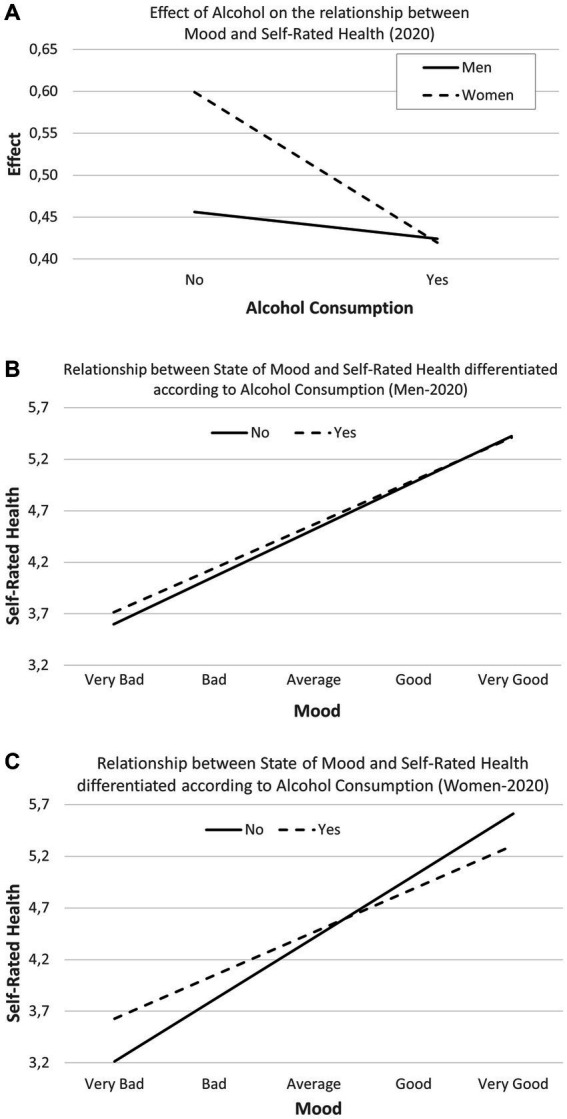
**(A)** Interaction effect between Mood × Alcohol × Sex on Self-Rated Health (first online COVID-19 survey, 2020). **(B)** Interaction effect of Alcohol Consumption and Mood in men on Self-Rated Health (first wave, 2020). **(C)** Interaction effect of the Alcohol Consumption and Mood in women on Self-Rated Health (first online COVID-19 survey, 2020).

Figures 4A–C show the interaction effect of Alcohol and sex on the relationship to Mood and Self-Rated Health in the first survey.

This study has analyzed how the relationship between Mood and Self-Rated Health is modified as a function of sex and Alcohol Consumption.

Figure 4A includes the interaction between Mood × Alcohol × Sex. The effects produced by Alcohol are greater for non-consumers than for consumers and for women than for men, with statistically significant differences.

The relationship between Mood and Self-Rated Health is positive, which implies that a low Mood is identified with a poor Self-Rated Health, and conversely, a high Mood means a very good health assessment. However, the study of this issue by disaggregating the population by sex and Alcohol Consumption reveals that for men Alcohol Consumption raises the score of the health assessment, being somewhat higher when the Mood assessment is low (Figure 4B). For women (Figure 4C), Alcohol Consumption improves their health appraisal when the Mood is low, but not in high Moods, where the opposite effect occurs.

**Figure 5:**
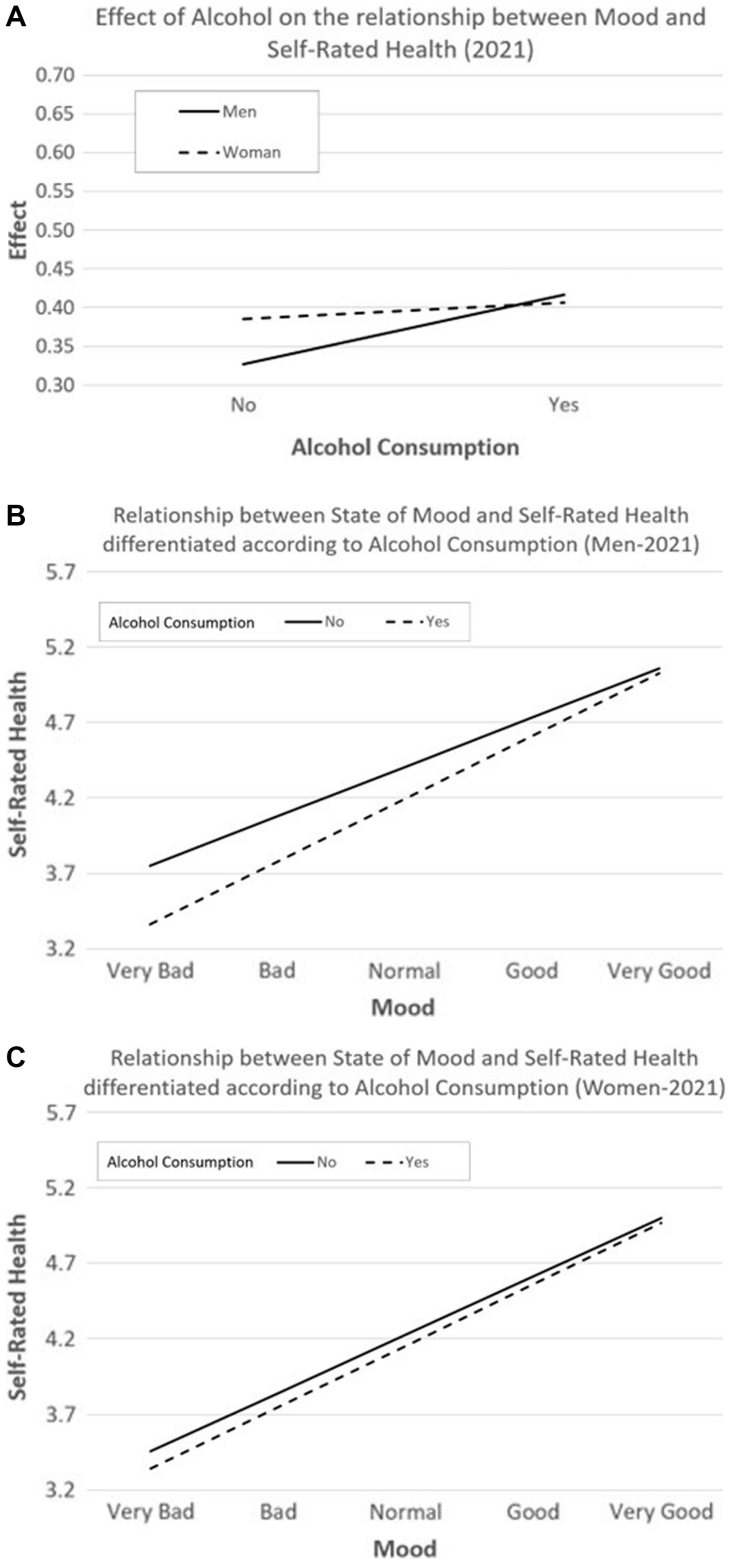
**(A)** Interaction effect of Alcohol Consumption and Mood on Self-Rated Health (second online COVID-19 survey, 2021). **(B)** Interaction effect of Alcohol Consumption and Mood and men on Self-Rated Health (second online COVID-19 survey, 2021). **(C)** Interaction effect of the Alcohol Consumption and Mood and women on Self-Rated Health (second online COVID-19 survey, 2021).

Figures 5A–C show the interaction effect of Alcohol and sex on the relationship between Mood and Self-Rated Health in the seventh wave of the COVID-19 pandemic (2021). No statistically significant differences were found.

## 4. Discussion

This study has revealed how the Spanish population residing in Zaragoza at two important moments in time during the COVID-19 pandemic [the first pandemic wave and seventh wave in Aragón (sixth wave in Spain)] adopted unhealthy behaviors in their lives as a coping strategy to deal with the new scenarios caused by the pandemic. As observed, the growth in the consumption of anti-anxiety medication, tobacco, Alcohol and online shopping mainly is noteworthy.

Regarding people’s health status, both men and women reported worse Self-Rated Health and Mood in the first survey, with an improvement in the self-perception of both items in the second survey. Furthermore, women reported worse results than men, both in Self-Rated Health and Mood, in the two periods analyzed.

Finally, moderation analyses showed that Alcohol only moderated the relationship between Mood and health in the first survey—conducted during the first wave—and that effect was lost in the second survey, which coincided with the seventh wave of COVID-19, when restrictive measures were no longer as severe.

The COVID-19 pandemic has had a major impact on the well-being of the general population (38, 39). People still suffer many consequences of the pandemic and will continue to do so for a long time, regardless of being COVID-positive or not. The general population understood and accepted the restrictive measures adopted to contain the pandemic and avoid the saturation of the healthcare system (40). Even so, most people reported a negative impact on their perception of their own health status and mood. As mentioned above, the causes for this are diverse: mobility restrictions, social isolation, employment, economic and family losses, etc. The results show that self-perception of health and mood was worse during the period of strictest confinement than in the second survey, which coincided with less restrictive measures and more social contact (41). Several articles in the scientific literature report the beneficial effects of social support, contact and maintaining work for well-being, mood and health (40, 42, 43). During strict confinement, relationships mediated through ICTs increased, thus demonstrating their effectiveness as an alternative for social support (44, 45). However, this type of relationship does not make up for the social distance and quality of face-to-face human interactions and their beneficial impact on health (46). Training workers about appropriate coping styles to adopt may be essential to enact prevention strategies to reduce the negative impact on the well-being in workers at a time of intense stress such as has occurred during the COVID-19 pandemic (43). In the same line, some studies have demonstrated that Resilience Strategies (Protective Practices) as spending time with family and friends are beneficial to worked wellbeing, mainly in turbulent working times with risk of job stability (47, 48).

The COVID-19 pandemic, like other diseases, has exacerbated existing gender inequalities (49), as shown in the results and in the two surveys conducted. Although from a biological perspective men were at greater risk and had more complications during the COVID-19 pandemic (50, 51), certain gender impact has also been observed. As is well-known in the literature and among the scientific community, women present worse Self-Rated Health than men, so it is a constant today (52, 53). Globally, women suffered more job losses than men. In Spain, according to data from the Labor Force Survey, unemployment rose more among women than among men (13.4% vs. 17.4% in men, women 8.9% vs. 13%) in the fourth quarter of 2019 (54). Likewise, home confinement had a direct impact on household care work, mainly performed by women. Many women during this pandemic have had to make real efforts to reconcile work, teleworking and family life, and this has had a direct impact on their health and well-being (51, 52, 55).

During the COVID-19 pandemic, many people have experienced significant mental issues (56). Unhealthy behaviors, such as consumption of anxiolytics, alcohol, tobacco and online gaming have been used as a coping strategy to deal with the situations caused by the pandemic (confinement, death, loss of employment, among others) (57). Drinking patters and associated harm are linked to diverse environmental factors such as physical availability of retailed alcohol, exposure to advertisements, and socioeconomic inequalities (58, 59). Bantounou (60) in their narrative review found that the predominant trend was a rise in alcohol consumption in approximately 20% of the population relationship with the health risk mentioned above (excess worry, stress, loneliness, poor mental health, lack of organization, female sex, young age, living in a big city a having child at home). On the other hand, the higher alcohol consumption observed in women in our study has been previously found in previous research. Studies conducted in previous crises such as 9/11 showed that there was an increase in alcohol consumption in women and not in men (61). Prior to the COVID 19 crisis, an increase in alcohol consumption, especially among women, was already being observed in Europe. Several factors have been associated with increased alcohol consumption during the COVID 19 pandemic. Most notably, women’s alcohol threshold requires less alcohol than men’s, a high percentage of women work, have children, and are responsible for informal care in the home, risk factors during the pandemic for consuming greater amounts of alcohol during times of increased stress such as extreme confinement. All of these risk factors were reduced during the second survey period, which may explain why our results changed and why these differences between men and women were no longer observed (62).

Many people have resorted to psychiatric medication to deal with stress caused by the pandemic—also known as coronaphobia—to reduce anxiety, depression and even insomnia (63). Along these lines, Santomauto et al. (64) reported that the pandemic has had a substantial impact on the risk of major depressive and anxiety disorders, estimating an additional 53.2 million cases of major depressive disorder and an additional 76.2 million cases of anxiety disorders globally due to the COVID-19 pandemic (64). All this explains the growth in the consumption of psychiatric medication that we have linked to this study.

The increase in alcohol and tobacco consumption that can be observed in the results is consistent with that described in the scientific literature (65, 66). Moreover, several articles show the increase in the online sale of alcohol and the rise in its consumption at home, together with the surge in tobacco use during the first wave of the COVID-19 pandemic, the period of strict confinement (67, 68). These consumptions were subsequently reduced, with the return to normality (19).

Finally, in the first survey, which coincided with the period of strict confinement, alcohol moderated the relationship between Mood and Self-Rated Health. This effect was lost in the second survey, when the restrictive measures were less severe. In addition, women who did not report drinking alcohol during strict confinement reported better results in Self-Rated Health and Mood than men and women who did report drinking alcohol. This is consistent with the European Alcohol and COVID web survey, in which 65% of the sample were women with a medium or high level of education—similar to our survey—and which revealed that during strict confinement alcohol consumption decreased or stabilized (69). However, the pattern of alcohol consumption has changed during the pandemic, shifting from public places to the home during strict confinement (70).

This study helps to understand the health behaviors that the population developed during two important moments of the COVID-19 pandemic. It also explores the moderating effect of alcohol on the relationship between Mood and Self-Rated Health. One of the strengths of this study is that it considers two moments of the COVID-19 pandemic: the first and second online surveys during the COVID-19 pandemic in Spain. Likewise, although the data cannot be generalized to the rest of the population, it does help to increase knowledge and to reflect on the measures to be adopted in the face of new situations of social isolation such as those that have occurred recently, mainly in vulnerable populations, with fewer resources and less capacity to cope. The main limitation of this study is its design (cross-sectional), as it cannot be used to establish causal relationships (1, 3). However, cross-sectional studies with surveys conducted on different populations and at different times are common in epidemiological analysis because, although they are not longitudinal, they serve to observe specific health outcomes and behaviors and establish measures from a public health point of view (2, 4, 5, 7). Another limitation is the type of non-probabilistic sampling used, which makes it difficult to generalize the results.

Although the samples showed no differences in sex and age, the fact that more than 50% of the participants were women with a high level of education is another limitation of the study. In a study carried out by the Sociological Research Center (CIS) in Spain, where different factors that could influence people when answering the surveys were analyzed, it was observed that in relation to gender, a determining factor in the participation was the interview theme. COVID and its consequences are closely related to the social problems with the greatest interest in women and their role as caregivers, a fact that could also justify their greater participation in the surveys analyzed in this study. In addition, a previous study that analyzed the different motivations for people to participate in online surveys, they concluded that there were important differences depending on the level of occupation of the respondent, since those with a high social class participated mainly because they were interested in the subject of the survey and because of the reward they were given for participating, a fact that was reflected as we have pointed out in the surveys analyzed in this study (71). It should also be noted that the age group with the highest participation in both surveys was the young population. It is consistent with the study carried out with the data and methodology of the 8th edition of the European Social Survey, which also showed that young people under 30 years of age were the most willing to participate in online surveys, mainly related to their regular use of new technologies (72). For all this, Zaragoza City Council, after observing the characteristics of the population participating in the two surveys, analyzed the characteristics of the samples with the census data available and observed that the characteristics were similar to those of the Aragonese society as a whole at the time of the study. Likewise, it is important to point out that the data are self-reported and should be treated with caution. Finally, another important limitation is that the study has considered whether people reported consuming alcohol or some other unhealthy behavior—such as the consumption of certain medications—but not the amount of alcohol or the number of pill units. As the data were collected using an online survey, interviewer bias has been controlled. Finally, and despite the limitations of this study, the results provide information consistent with the literature reviewed, which shows that in periods of confinement or restriction as a consequence of pandemics such as COVID-19, the development of negative attitudes toward health such as alcohol consumption can influence Self-Rated Health and Mood.

The COVID-19 pandemic has had a considerable impact on our lives and our social and health systems. During the period of strict confinement, people’s levels of stress and instability rose due to the uncertainty generated. Many people suffered consequences to their health and, more specifically, to their psychological health. This study has revealed the development of unhealthy behaviors as a strategy to cope with the situation, such as an increase of alcohol and tobacco consumption at home. All this should be analyzed and considered from the perspective of public health. New studies are needed to address the social thresholds of alcohol consumption, taking into account different perspectives, including environment-specific prevention, as well as understanding variations in the intrapersonal and social perception of drunkenness, as this has been shown to be inconsistent across cultures and time periods (58).

Future work must focus on health promotion strategies and the ability to adapt to uncertain scenarios that affect people and their resilience. The population must learn to adapt to and overcome harsh scenarios such as those experienced during the COVID-19 pandemic, since globalization and climate change make us vulnerable and, in all probability, we will have to face future pandemics. We must learn from the past to improve in the future.

## Data availability statement

The datasets presented in this study can be found in online repositories. The names of the repository/repositories and accession number(s) can be found at: “Encuesta Condiciones de vida, percepcion y valoracion emocional de la ciudadanía durante el confinamiento” conducted by the Zaragoza City Council, available at https://www.zaragoza.es/sede/portal/datos-abiertos/servicio/catalogo/2400; and “II Encuesta Condiciones de vida, necesidades y expectativas tras 20 meses de pandemia por Covid-19” conducted by the Zaragoza City Council, available at https://www.zaragoza.es/sede/portal/coronavirus/encuesta.

## Ethics statement

The Zaragoza City Council was responsible for obtaining informed consent. The studies were conducted in accordance with the local legislation and institutional requirements. The participants provided their written informed consent to participate in this study.

## Author contributions

RS-R: Conceptualization, Data curation, Formal analysis, Investigation, Methodology, Software, Supervision, Validation, Visualization, Writing – original draft, Writing – review & editing. JP-H: Conceptualization, Data curation, Formal analysis, Methodology, Supervision, Writing – original draft, Writing – review & editing. ÁA-M: Data curation, Writing – original draft, Writing – review & editing. SV-P: Supervision, Visualization, Writing – review & editing. MZ-A: Funding acquisition, Resources, Supervision, Visualization, Writing – review & editing. MC-B: Supervision, Visualization, Writing – review & editing.
